# Extracts and Gleanings

**Published:** 1871-06

**Authors:** 


### PART II.

### Abridged Extracts and Gleanings from our Exchanges

*MULTUM IN PARVO.*

*Epilepsy Treated with Hydrate of Chloral.*—Dr. Louis Mackall, Georgetown, D. C., reports two cases in the National Medical Journal, of epilepsy "controlled and subdued" by the administration of hydrate of chloral. The second case had been treated by almost every known remedy, without benefit, for two years, when seen by Dr. Mackall. "He began with 10-grain doses of chloral, at night, and from the time of his first taking it to the present, an interval of more than seven months, he has never had a fit, and his nights have been passed in quiet sleep"—gaining "strength both mentally and physically." He calls attention to the fact that "unlike other anodynes, the dose of chloral does not require to be increased."

The editors in a note call attention to two other cases greatly benefitted by the use of chloral, which cases were reported by Dr. Beauchamp, in the American Journal of Medical Sciences, of April, and by Dr. F. K. Bailey, of Knoxville, Tenn., in the Med. and Surg. Reporter. Dr. Bailey had used unsuccessfully the bromide of potassium before giving the chloral.

*Aneurism cured by hypodermic injection of Ergotine.*—Dr. Jacobson, of Copenhagen, records the successful employment of hypodermic injection of ergotine in two cases of aneurism. In one case injections of an aqueous solution of secale were made in the vicinity of the tumor, causing it to disappear in eight days, having existed twenty years. This mode of employing secale for the cure of aneurisms is worthy of trial in all suitable cases.

*Nux Vomica for Biliousness.*—Dr. J. M. Scudder says: "In these cases I have prescribed nux vomica with most decided success. If it is a case of fever or inflammation, the remedy is alternated with the special sedatives, and in place of preventing their action as many might suppose, it seems to favor it. It is continued until these unpleasant symptoms pass away—one, two or three days. I order it in the following proportion:

℞—Tr nux vomica, . . . gtts. x to xx.
Aquæ, . . . . ℥ iv. M.

S. A table-spoonful every one or two hours.—*Eclectic Medical Journal.*

*Dropsy.*—Dr. Scudder extols the following formula for dropsy:

℞—Tr. apocynum, . . . . gtts xx to ʒ ii.

Aquæ, . . . . ℥ iv. M.

S. Teaspoonful every two or three hours. In the dropsies following hard drinking he uses:

℞—Tr. Nux vomica, . . . . gtts xv.

Tr. apocynum, . . . . ʒ ss.

Aquæ, . . . . ℥ iv.

S. Teaspoonful every three hours.—*Eclectic Med. Journ.*

*For Neuralgia.*—The following formula is highly recommended in "obscure neuralgic difficulties:"

℞—Prussiate of Potash, . . . . ʒ iv.

Simple syrup,

Water, aa, . . . . ℥ ii. M.

S. A teaspoonful three times a day.—*Eclectic Med. Journ.*

*Tetanus successfully treated by hydrate of chloral,*—Dr. W. B. Cluness gives in detail, in the Pacific Medical and Surgical Journal, the successful treatment of a case of tetanus with hydrate of chloral. By its use the pulse was reduced from 128 to 80. The successful issue of this case will justify the employment of chloral in tetanus. Dr. George Thompson, also, in the Richmond and Louisville Medical Journal, reports a case of supposed idiopathic tetanus cured by chloral. The dose was thirty grains, repeated several times a day. The cure was effected in four or five days.

*Wakefulness.*—Dr. Wm. A. Hammond (Detroit Review of Medicine and Pharmacy,) recommends *phosphorous* for wakefulness. Twelve grains of phosphorous are boiled in an ounce of almond oil and filtered. Half an ounce of this is mixed with an ounce of gum arabic, and fifteen drops of some aromatic oil are added. Of this mixture the dose is 15 drops—containing one-twenty-fourth of a grain of phosphorous. Three doses are given before bed-time, generally producing sleep on the second day if not on the first. The dose may be increased one drop a day, until twenty drops are taken, or signs of gastric irritation supervene. He has also employed for this purpose the bromide of potassium combined with *sumbul.* The dose of the fluid extract of sumbul (Neergaard's) is from 20 drops to a drachm three times a day.

*Epilepsy treated successfully by Assafœtida.*—Dr. Pollock, of Charing-Cross Hospital, reports a case of epilepsy (Lancet) cured by assafœtida, after the failure of bromide of potassium, sulph. zinc, quinine and tr. iron. A drachm of tr. assafœtida, with three grains of carb. ammonia, were given three times a day—resulting in a cure in a few weeks.

*Diabetes treated bg carbonate of ammonia.*—Dr. Pavy states that the carbonate of ammonia (100 grains dissolved in a pint of water, and all taken during the course of twenty-four hours,) exerts a controlling influence over diabetes. Dr. A. S. Dunkin has employed the milk diet in this disease with great success. The treatment should be *methodically persevered in*, by confining the patient exclusively to the milk treatment—i. e. skimmed milk being used alone as a diet to the exclusion of every other article. It is necessary in adopting this treatment to commence with small quantities of milk—say half to one glassful three times a day, gradually increasing the quantity as the patient becomes accustomed to its use.

*Carbolic Acid in Scarlet Fever.*—Dr. Cleaver (Iowa Medical Journal,) has used the following formula with decided advantage in this disease:

℞—Carbolic acid, . . . . ʒ ij

Alcohol, dilute . . . . ℥ ij. M.

S. Mix a teaspoonful of this to a tablespoonful of water, used either as a gargle or with a mop, depending upon age and ability to gargle—say once every two hours. Also give ten to twenty drops of same in muc. g. acacia at same intervals—dose depending upon age of patient. This treatment was employed in 70 cases, with marked success.

*Corrosive Sublimate for Improving the Constitution.*—Dr. Almes (L'Union Medicale,) has used corrosive sublimate largely in adults and children for building up the powers of the system, with decided advantage. He has continued its use for half years and whole years, the patients constantly improving—appetite returning—increase of bulk, fat, colororation of complexion, development of muscular vigor, and all the external signs of health. The dose given for this purpose was very small—given dissolved in distilled water.

*Epithelioma successfully treated by chlorate of potash.*—Prof. Magni, of Bologna, after using nitrate silver and acid nitrate of mercury unsuccessfully in one case of this cancroid affection, employed the chlorate of potash externally and internally, which effected complete cicatrization at the end of a month.

*Prophylactic action of Copper in Cholera Epidemics.*—From the investigations of Dr. Clopton and M. Burq, it it announced that persons employed in working copper escape cholera and the choleracic diarrhœa. By analogy, copper might be found useful as a prophylactic in epidemics of cholera morbus, diarrhœa and dysentery.

*Internal Administration of Chloroform.*—Dr. G. W. Murdock, in the Journal of Pharmacy, after alluding to the difficulties physicians have in finding a good vehicle for the internal administration of chloroform, recommends *pure glycerine* for the purpose. In preparing it he takes one part of chloroform with two parts of glycerine (best) add the chloroform very slowly, and rub up carefully. Then put in a bottle, and let it stand 24 hours. A little chloroform will have deposited at the bottom. Separate this, and rub it up with the third part of glycerine, then mix it with the rest and the solution is complete. Six ounces of glycerine with two ounces of chloroform will give seven ounces of the solution so that each fluid drachm contains about 17 minims of chloroform. By adding morphia at time of administration this will be found a valuable antispasmodic for colics, etc.

*Cundurungo.—A new remedy for Cancers, Scrofula and Venereal Diseases.*—The National Medical Journal contains a diplomatic correspondence relative to the reputed virtues of "the wood, condurango," specimens of which have been forwarded to the navy department by the Government of Ecuador for analysis and experiment. Resident physicians of Quito, Ecuador, extol it as a "cancer specific," and valuable alterative and restorative in scrofula and venereal diseases. Should it prove to be half as valuable as represented, the medical world will have another remedy for these diseases. In this instance, as in other remedies declared to be specific, we say to the profession, "prove all things ; hold fast to that that is good."

*Propylamine—a remedy for acute and chronic Rheumatism.*—Dr. J. R. Nichols (Boston Jour. of Chem.) remarks that this remedy "has continued to grow in favor during the past 12 years, and some physicians regard it as almost or quite a specific in acute and chronic rheumatism." "Dr. Awenarius, of St. Petersburg, treated 250 patients in the Hospitals of Kanlinkin, and in acute cases the pain and fever *always disappeared the next day.* He regards it as a true specific for the various affections of rheumatic origin." "The remedy is prescribed in the following manner:

℞—Propylamine, . . . gtts. xxv

Aquæ distil. . . . f℥ vi. M.

When necessary, add

Aq. saach. peppermint,. . ʒ ii.

S. A tablespoonful every two hours.

*Growth of the Nails as a prognostic indication in Cerebral Paralysis.*—Dr. S. Weir Mitchell (Trans. College Physicians—Am. Jour. Med. Sciences) gives a number of cases to corroborate the proposition that the growth of the nails of the fingers of a paralyzed arm is retarded during the time the limb is paralyzed. The nails on the sound side grow as usual, while those on the diseased limb remain stationary, and do not begin to grow until there is a return to a healthy condition of the paralyzed limb. He hopes "that this apparently trifling symptom may open the way to the solution of very important questions, and is certainly not devoid of interest for the most purely practical among us."

*Chronic Eczema.*—Dr. Thomas Barrow, Baltimore, Md., gives (Med. and Surgical Reporter,) several cases of chronic eczema which he permanently cured by the following plan of treatment:

℞—Tinct. ferri chlor. . . . . f ℥ iij.

S. 20 drops before each meal, as an ectropie and to remove anaemia.

℞—Acid nitro chlor. . . . . ℥ i.

S. Six Drops, five minutes after each meal, as an eliminative and ectropic.

℞—Cupri sulph. . . . . . ʒ i.

Aquæ, . . . . . f. Oi. M.

S. Apply to the whole diseased surface once every other day, as a local alterative. These remedial agents are capable, he asserts, of entirely and permanently curing this obstinate malady within about one month.

*Acute Rheumatism.*—Dr. J. T. Davis (Cincinnati Med. Repertory,) gives the following treatment for rheumatism: When called to a patient with the disease, he first orders him to be placed between blankets, and if suffering much, gives one or two grains of opium. He then proceeds with the following, administered alternately every three hours:

℞—Quiniæ sulph. . . . . gr. xx.

Potass. nit. . . . . ʒ i. M.

ft. chart No. 6.

S. One powder every three hours.

℞—Tr. aconite,

Tr. colchici, aa, . . . . f. ʒ i.

Potass. bicarb. . . . . ʒ iss.

Aquæ, . . . . ℥ vj. M.

S. One tablespoonful every three hours.

He has found, in a large number of cases the above treatment to have been excellent. He gives plenty of milk and good nourishing food. The bowels must be kept open.

*Relief of Pain in Otorrhœa.*—Dr. Post relieves the pain of otorrhœa—ear-ache—by applying a leech to the helix. He regards it as the surest and best means of affording relief.

*Codeia and oxalate of cerium in Dysentery.*—Dr. J. W. Moore reports in the proceedings of the Medical Society of the county of Albany, N. Y., (Buffalo Med. and Surg. Journal,) the great value of codeine in dysentery. He first used it for this purpose three years ago. All the other remedies had been tried that could be thought of with no apparent relief. There was obstinate vomiting, bloody and mucus stools, with bearing down pains and tenesmus. The patient was unable to take any nourishment—small quantities of water being immediately rejected by the stomach. Opium by injection was tried, which increased the nausea and induced headache. At last two grains of oxalate of cerium was dropped dry upon the tongue. The retching ceased so that in the course of an hour the patient retained a grain of codeia in solution, followed by sleep which lasted several hours. The headache was relieved. A fæcal stool followed the administration of sulph. magnesia. Codeine and cerium was continued several days, and the patient soon recovered. He has used this treatment in fifteen or twenty cases, and asserts that persons are relieved of dysentery by this remedy when the stomach can retain it. He has never given more than two grains at a dose, and has never observed any unpleasant effects when the medicine was pure.

*Diptheria treated by Quinine.*—Dr. R. C. Russ, Hillsboro', O., (Cincinnati Lancet and Observer,) says: "A great deal depends upon the early administration of quinia; in this disease it is a *sine qua non.* I have frequently seen this disease yield as readily to large doses of quinia as intermittents or remittents, when given within the first twelve or twenty-four hours.

*Croup treated by the vapor of Slacking Lime.*—Dr. J. H. H. Burge, Brooklyn, N. Y., (New York Medical Journal,) claims great success over every other method of treating croup by using the vapor of slacking lime. A few pieces of lime are put in a pitcher and warm water poured on it. A blanket or a newspaper is thrown over the pitcher so as to direct the vapor towards the mouth of the patient and compel him to breathe as much of it as he possibly can. This is to be kept up continually day and night so long as the disease lasts, only stopping, in severe cases, to drink punch or beef tea. When the disease abates, the lime may be slacked in a pail placed at the side of the bed. Some otherwise hopeless cases were cured by this plan of treatment.

*Hydrate Chloral in Singultus.*—Dr. T. L. Leavitt, Germantown, Pa., reports (Am. Jour. Med. Sciences,) three cases of obstinate hiccough relieved permanently by 5-grain doses of hydrate of chloral, after the failure of ether, bromide potassium, musk, camphor, &c.

*Tape-Worm expelled by Bromide of Potassium.*—Dr. J. N. Northrop, (Buffalo Medical and Surgical Journal) after employing spts. turpentine, pumpkin seed, etc., in a case of tape-worm, gave the bromide of potassium in 20-grain doses every four hours. The patient continued the remedy two and a half days, then took half an ounce of spts. turpentine, and in a short time two ounces of castor oil. He passed 200 feet of worm. After one week repeated the medicine and passed 50 feet more, with the head. Dr. Northrop also gave the bromine of potash in syrup, to a child two or three years old, in sedative doses, when twelve feet of tape worm were passed.

*Idiopathic Anœmia.*—Prof. Sam. S. Armor (Medical and Surgical Reporter,) in cases which he calls "idiopathic anæmia, where there is headache, palpitation of the heart, nervousness and general weakness—in females, uses the following formula :

℞—Iron by hydrogen, . . ʒ iss.<br>

Quin. sulph., . . . ʒ j.<br>

Arsen. acid, . . . gr j. M.<br>

ft. pil No. 50.

S. One after each meal. He orders, in addition, a pill composed of one-half grain each of watery extract of aloes and hyosciamus ; one to be taken after dinner to keep up gentle eliminative action along the intestinal track.

*Graunlar Lids and chronic Opthalmia.*—Dr. John Williams publishes the following formula, which after long experience, he highly recommends :

℞—Arsenici sulphureti . . gr ij.<br>

Unguenti citrini, . . . ʒ ij.<br>

Axungiæ preparat. . . . ʒ j. M.

In cases of granular lids, panmus and obstinate chronic opthalmia, the upper eyelid should be everted, and a piece the size of a flaxseed he applied with a camel-hair pencil into the superior palpebral sinus.

*Arsenic in Dyspepsia.*—Dr. Thorowgood finds arsenic valuable in some forms of dyspepsia. One drop of Fowler's solution given in half an ounce of infusion of calumbæ, in one case, allayed the pain and vomiting of food, and enabled the patient to eat and digest small quantities of mutton. In cases where the tongue is small and irritable with projecting papillæ and coated by a grey or yellow fur, arsenic will do good.

*Chloral in rigid Os Uteri.*—Dr. S. F. Starley (Am. Practitioner,) speaks highly of the value of chloral in producing relaxation in cases of rigidity of the os uteri. He thinks it should not be used if organic disease of the heart exists.

*Hypodermic doses of Morphia.*—Dr. E. T. Wilson says a quarter of a grain of morphia is, as a rule, an unsafe dose to commence with. A third of a grain has caused dangerous symptoms in 15 minutes, and in a case of mania half a grain has proven fatal. The injected fluid should always be thrown under the skin very gradually and slowly.

*Treatment of Lumbago.*—Dr. Sam. Wilks, (Lancet,) says cases of acute or recent lumbago are generally amenable to warm stimulating applications, as hot air, steam, fomentations, hot water and tincture of capsicum. It is very intractable when it becomes chronic. He has found benefit from subcutaneous injections of morphia and galvanism.

*Local Application to Burns.*—Dr. Binikerd (Med. and Surg. Reporter,) applies to burns, when seen early, carbolic acid and glycerine in the proportion of from 5 to 10 drops of the former, to ℥ ii of the latter. This is spread upon the burn by camel-hair pencil or feather, then a layer of white cotton retained by a roller bandage. For the suppurative stage, he employs the following :

| | |
|---|---|
| ℞—Yellow wax melted and strained, . | ℥ i. |
| Raw linseed oil, . . . | ℥ iij. |
| Tannin, . . . | ʒ j. |
| Sub nitrate bismuth, . . . | gr xx. M. |

First melt the wax, add the oil, and stir the whole; then add the tannin, and lastly the bismuth. The ointment should be applied on pieces of lint.

*Glycerate of Tar.*—Mr. J. B. Moore gives the following formula :

| | |
|---|---|
| ℞—Picis liquidæ (strained,) . . , | ℥ j. (troy) |
| Magnesiæ, carb. (rubbed to powder on a seive,) | ℥ iij. |
| Alcoholis, . . . . | ℥ ij. |
| Glycerine, . . . . | ℥ iv. |
| Aquæ, . . . . | q. s. |

Mix the alcohol and glycerine with ten fluid ounces of water. Put the tar in a mortar first with the carb. mag. gradually added, until a smoothe pulverulent mixture is obtained; then gradually add, in small portions at a time, with thorough trituration continued for fifteen or twenty minutes, six fluid ounces of the mixture of alcohol, glycerine and water, and strain, with strong expression ; return the residue to the mortar, and repeat the trituration as before, with five fluid ounces more of the same liquid and express; again treat the dregs in the same manner with the remainder of the menstrum, and after expression reduce the residue by trituration to a uniform condition, and finally pack firmly in a glass funnel prepared for percolation, and pour upon it the expressed liquors previously mixed, and when the mixture has all passed from the surface, continue the percolation with water until one pint of the liquid has been obtained.

In conjunction with the fluid extract of wild cherry-bark, acetate or syrup of squills, syrup of guinaria, lactunus carium, etc., in varied proportions to suit the prescriber, it will form elegant and palatable combinations, adapted to the treatment of chronic coughs and the various diseases of the pulmonary organs. The dose of the glycerate is from a desert to a tablespoonful.

*Suppurative Hepatitis successfully treated with Muriate of Ammonia.*—Dr. L. G. Alexander, Ky., communicates (Med. and Surg. Reporter,) a case of hepatitis successfully treated by the above medicine. He found the patient with high fever, complaining of dull, heavy pains in the right hypochondriac region ; there was enlargement of the liver and dullness upon percussion. The patient was unable to lie on left side, and pain increased by pressure or cough of the organ. He had been having intermittent fever. After treating the case ten days with the usual remedies, and the patient growing worse, he then gave the following prescription, under the influence of which he at once and rapidly improved :

℞—Muriate ammonia. . - grs v.
Tr. sarsaparilla, . . ʒ ii. M.

S. This quantity to be taken every four hours.
Also : Pulv. Doveri, . . gr. iii.

S. Every four hours. Milk punch and beef tea ; bran poultice to bowels. The good effects of the ammonia were evident from the second dose, increasing the amount of urine, and acting on the secretive system generally.

*Chlorosis.*—Prof. S. G. Armor (Med. and Surg. Reporter,) in cases of chlorosis, where there is deficient menstruation, constipation of the bowels, flatulency, and great nervousness, employs the following formulæ :

℞—Arsenious acid, . . . grs iv.

Acid hydrochlorici, . . ʒ iss.

Mur. tinct. iron, . . ℥ iiss.

Aquæ ad. . . . Oj.

S. Teaspoonful after each meal.

℞—Ext. aloes, aqueous, . . gr xx.
Sulph. iron, . . gr ix.
Ext. nux vomica, . . gr v. M.
ft. pil No. 20.

S. One pill to be taken after dinner, or two a day, if necessary.

*New Operation for Entropium.*—James McCrath, Surgeon to the British Hospital, Smyrna, had devised the following simple operation for entropium, with much success. Turning up the eyelid, he makes a horizontal incision parallel to the border, and about on a level with the seat of the bulbs, including the whole extent, or very nearly, from one canthus to another, avoiding the duct at inner canthus. He then makes another incision parallel to the first, removing a narrow strip of the cartilage, less than the twelfth of an inch in breadth.

*Turpentine in Infantile Convulsions.*— When convulsions continue in colicky children, and are not relieved by the warm bath, injections of warm water, brandy or chloric ether with an alkali, Dr. Graves recommends turpentine to be given.

℞—ol. terebenthi, . . . ʒ i.

ol. ricini, . . . ʒ iv.

Mist. acaciæ,

Aq. cinnamon, aa, . . . ℥ iij.

S. Teaspoonful every three hours. This acts on the bowels and produces a copious discharge of urine.

*Neuralgia.*—A correspondent of the Lancet says: "A few years ago, when in China, I became acquainted with the fact of the natives, when suffering with facial neuralgia, using oil of peppermint, which they lightly applied to the seat of pain with a camel hair-pencil. Since then, in my own practice, I frequently employ this oil as a local anæsthetic, not only in neuralgia, but also in gout with remarkable good results."

*Intussusception treated by Injections of Warm Water.*—Dr. Wm. Faulkner gives two cases (Med. and Surgical Reporter,) treated as above. He places the patient upon an inclined plane, head down, and fills the lower bowels to distention with warm water. Both cases recovered. He suggests the employment of full hypodermic injections of morphine before using warm water.

*Syphilitic Ulcers.*—Prof. S. D. Gross, in surgical "clinic remarks," (Med. and Surg. Reporter) says: "Whenever you get these multiple sores, vesicular or pustular, occurring in the extremitles, and particularly if they are obstinate and resist treatment, you may put them down as specific." In such cases he gives the following:

℞—Potats. iodid. . . . gr viij.
Hydrarg. chlor. cor. . . gr 1–10
Pulv. iodidi, . . gr 1–10 M

S. This is to be taken three times daily. Locally he treats these sores with dilute nitric acid, one part to five, every fourth day.

*Antagonistic Powers of Opium and Belladonna.*—Dr. John A. Little, Ohio, in a lengthy article (Med. and Surg. Reporter), gives many cases of his own and that of others, corroborative of the antagonism between opium and belladonna. He remarks: "Many other cases are on record, but these I believe to be sufficient to demonstrate conclusively the mutual antagonistic properties of these two medicines. If this is true, it is a matter of importance that it should be fully appreciated by the profession." "There seems to be among the profession some degree of timidity in regard to the use of belladonna. It is apt to be looked upon not only as a very potent, but also as a very dangerous remedy. Compared with opium I believe it quite safe. The poisonous effects of opium come on so insidiously, and their approach is so graduul, that they are often not detected until there is complete narcotism. It is not so with belladonna; the largely dilated pupil and the erythematous rash give warning in time." "It must be constantly borne in mind that poisonous doses of one must be met with equally poisonous doses of the other, to be successful. It is clearly proven that one which in ordinary circumstances would be poisonous, is not only innocent but salutary when there has been a poisonous dose of the other taken."

*Hydrochlorate of Apomorphia in Rigid Os.*—Dr. Milne gives a case of extremely rigid and unyielding os in which immediate relaxation was caused by one-fourth grain of apomorphia. This drug is an energetic and powerful emetic.

*Diarrhœa of children.*—Dr. C. C. Fuller says when the motions are of grass green color and slimy, one drop of the wine of ipecac, given every hour or less frequently, according to the symptoms, will usually result beneficially. When nausea and sickness of stomach is present, it furnishes an indication for the treatment.

*Ether in Nervous Flatulence.*—Ether has been found by Sir Fred. Pollock to afford great relief in his own person when suffering from gastric flatulence and painful spasms occurring after meals. It is inhaled from an ordinary bottle held to one nostril.

*Toothache.*—Dr. N. N. Hays (Boston Journal of Chemistry), states that he has had remarkable success in relieving toothache and neuraigia pains of the gums and face by the following:

℞—Chloroform, . . . . ʒ ij.

Tr. aconite rad. . . . . ʒ iss. M.

For simple toothache, a pellet of cotton wet with the liquid and inserted in the cavity of the tooth is sufficient. For neuralgic pains paint the parts affected, and relief is afforded. Tincture of aconite must be used with care, as it is poisonous.

*Purgatives in Skin Diseases.*—Dr McCall Anderson says: Purgatives or aperients are of service in at least one-fourth of all skin-diseases, at the outset at least, and many can be cured by the exclusive use of them. This is especially true of eczema, when associated with digestive disorders and constipation. If there be, however, great debility or ulceration, great caution is required in the use of purgatives. He uses the following formulæ:

| | | |
|---|---|---|
| ℞—Sulphate magnesiæ, | . . | ℥ iij. |
| Dilute sulph. acid, | . . | ℥ iss. |
| Sulph iron, | . . | ʒ iij. |
| Simple syrup, | . . | ℥ vj. |
| Infus. quassia ad | . . | ℥ xxiv. M. |

S. A table-spoonful in a good deal of water three times a day.

Another very useful tonic, aperient, especially if the bowels are easily moved, and if there are indications of nervous debility, is the following:

| | | |
|---|---|---|
| ℞—Phosphate soda, | . . . | ℥ iij. |
| Dilute phosphoric acid, | . . | ℥ ij. |
| Syrup ginger, | . . | ℥ vj. |
| Infus. gentian comp. | . . | ℥ viij. |
| Distilled water, to | . . | ℥ xxiv. M. |

S. A tablespoonful in a large wineglassful of water three times a day. Shake the bottle before taking.

*Tea-Leaf Poultices for Burns.*—Dr. W. H. Searles, in the Chicago Medical Examiner, recommends poultices of tea-leaves, moistened with hot water as preferable to all other remedies in the first stage of burns and scalds.—*Med. Gazette.*

*Curative effects of Pregnancy on Prolapsus Uteri.*—M. Brachet relates some cases in proof of the fact that a cure of prolapsus uteri may not unfrequently be procured, if the patient fall pregnant again and then be confined to her bed for a period not less than forty days after delivery.

*New Emmenagogue.*—Dr. Prescott (Med. Gazette, N. Y.) mentions a drug of great repute as an emmenagogue, which he had used with good success himself and had heard good accounts of from others. It was a Chinese drug called Tsa Tsin, Rhynchosus Excavata. The penalty for exporting it from China was death. Dr. Williams, who was travelling in China, contrived to bring some away, and had used it in fifteen hundred cases in Hospital practice.

*Post Partum Hemorrhage.*—Dr. Tyler Smith says, post-partum hemorrhage would be of seldom occurrence if ergot were given in full doses immediately and habitually at the moment of birth.

*Secondary Syphilis.*—Dr. S. R. Nissley, Pemberton, O., (Jour. Materia Medica,) has employed the following formula with uniform success:

| | | |
|---|---|---|
| ℞—Hydrarg. chloridi corrosivi, . | | gr. ij. |
| Potass. iodidi, . . . . | | ℥ ss. |
| Fl'd. ext. Taroxaci, . . | f. | ℥ ii. |
| " " Stillingia comp. . | f. | ℥ ii. |
| " " Sarsaparill. . . | f. | ℥ i. |
| Syrp. aurantii, . . . | f. | ℥ i. M. |

S. Teaspoonful three times a day.

*Intermittent Fever.*—Dr. S. C. Lacy, Pa., gives the following plan of treatment of intermittent fever (Jour. Materia Medica):

℞—Gelseminum Fl'd. Ext. Liquor potass. arsen. aa, ℥ i.

S. Twenty drops three times a day.

He says he has treated a large number of cases, and never knew a patient to have more than one chill after commencing the medicine. He continues the medicine for one week, omits five days, then gives it two days, and omits five days again. This is done four weeks, at end of which time the medicine is discontinued, and patient cured.

*Diabetes.*—Dr. F. S. Tumer thinks the following formula (Jour. Materia Medica,) almost a specific in diabetes. He has used it for thirty years, treating one hundred and nine cases with only three failures. If his diagnosis has been correct, this is indeed "almost a specific:"

| | |
|---|---|
| ℞—Syrup of Plaintin, . . | ℥ iv. |
| " Yarrow (ach. mille) . | ℥ iiiss. M. |

S. Four ounces once in four hours, to be continued for thirty days. Animal diet should be rigidly enforced, and bowels kept open by rhubarb.

*Convulsions caused by blows upon the Head.*—Dr. Hart (New York Med. Gazette) reports a number of cases of convulsions occurring in children and adults after receiving blows upon the head with the treatment employed by him in such cases. The first case was a little child six months old, who received a severe blow upon the head. Three days afterwards it became feverish and restless, and shortly thereafter passed into convulsions. A physician was called in who gave some medicine, and applied blisters behind the ears. He found the child in a convulsion when he arrived. One leech was applied to the temple, and large doses of bromide of potassium given—about 18 grains in twenty four hours. No more convulsions occurred, and in 36 hours the child was well. Two other cases of children were given—leeches were applied with prompt relief.

*Corrective Influence of Bromide of Potassium over Opium.*—Dr. J. M. DaCosta (Amer. Jour. Med. Sciences,) speaks highly of the great benefit derived by using bromide of potassium before giving opium in those patients in whom the latter drug produce unpleasant after-effects. He gives several cases illustrative of its action, in this respect proving its great utility and happy results. The bromide does not destroy either the anodyne or the hypnotic effects of the opiate: on the contrary, it rather heightens both, and more particularly the latter. He thinks the bromide acts best when given some hours before the opium, and forty to sixty grains—generally forty grains—prove sufficient.

*Cancrum Oris.*—Dr. John Burton, Ind. recommends the following for cancrum oris:

| | | |
|---|---|---|
| ℞—Cupri sulphas, | . . . | ʒ ss. |
| Pulv. cinchonæ, | . . . | ʒ i. |
| Aquæ, | . . . | ℥ i. M. |

S. Apply with camel-hair brush three times a day. Dr. Burton has tried this for twenty years without failure.

*Sub-nitrate Bismuth in Cholera Infantum.*—Dr. John Lessemain (Med. and Surg. Reporter,) states that in the majority of cases of this disease, it has acted for him as much the part of a specific as quinine does in ague. For a child two years old, he gives from four to eight grains every hour until the discharges are somewhat subdued, when the interval is lengthened. Occasionally it becomes necessary to give double that quantity. If there is much pain, he gives a twelfth or a tenth of a grain of opium with each dose. Milk and lime water are given in the interval. Externally counter-irritation is kept up by mustard to the epigastrium and extremities.

*Bromide of Iron in Involuntary Seminal Emissions and Spermatorrhœa.*—Dr. N. H. Norris, Beloit, Wisconsin, (North Western Medical and Surgical Journal), calls attention to the bromide of iron in these diseases, for which he has found it as much a specific as quinine is for intermittent fever. He gives from three to five grains rubbed up in a little simple syrup, to be taken one hour before or after meals, three times a day, and a sufficient amount at bed-time to procure good, refreshing sleep. Ten grains will usually accomplish this result, but he has given 20 grains without injury. He thinks it of *vast* importance to have the patient sleep. Combined with the bromide of iron, he employs cold ablutions to the genitals, bathing the parts night aud morning, together with moderate exercise. He thinks failure impossible, if the treatment is faithfully carried out.

*Burns and Scalds.*.—Dr. A. H. Lamphead (Boston Journal of Chemistry,) gives two applications for burns, which, in his hands, have frequently afforded almost instant relief to pain and preventing the separaton of the cuticle in cases where a blister seemed inevitable. The first is to envelope the injured part immediately with the pulp of the *raw irish potato.* The second is to apply linen cloths dipped in a mixture of *sweet cream and sub nitrate of bismuth*, in the proportion of one ounce of the latter to a pint of the former, repeated once in two or three hours.

*Chorea treated by ether spray to the Spine*.—Dr. John Rose reports (Lancet) the case of an anœmic girl, aged thirteen years, who had had rheumatism, but without heart affection, who was successfully treated by this means. The spray was applied to the spine, four or five moments each time, and after fifteen settings, a very marked improvement took place, followed by complete recovery.—*Medical News.*

*Iron in miasmatic diseases.*—Dr. S. P. Crawford, of California, in an article in the Nashville Journal of Medicine and Surgery, under the above caption, highly recommends the following formula for intermittent fever :

| | | | |
|---|---|---|---|
| ℞—Sub. carb. iron, | . . . | ℥ ss. | |
| Sulph. quinine, | . . . | ʒ ss. | |
| Simp. syrup, | . . . | ℥ vi. | M. |

S. Teaspoonful three times a day. Some of his patients did not have a chill after taking the remedy.

*Granular Lids.*—Dr. L. Tait says simple syrup, combined in some cases with other remedies, such as vinum opii or atropia, answers admirably. About three drachms of the best sugar to the ounce of water, filtered quite clean, forms a solution which does not deposit sugar. A little may be dropped into the eyes as often as they feel uneasy.

*Poultices.*---In acute lumbago no method of treatment is so sure and speedy as poulticing. The severest cases are greatly benefitted in a few hours, and generally cured in one or, at most, two days. The poultices must be very hot, large enough to cover the whole loins or part affected, and thick enough to remain quite hot for half an hour, when they should be changed. Continue for three hours, or longer if relief is not obtained; and when discontinued the parts should be wiped perfectly dry, covered with flannel, and this again covered by oiled silk. Our author prefers poultices made of bread.---*American Practitioner.*

*Neuralgia accompanying Shingles.*---Dr. McCall Anderson asserts that this form of neuralgia is best relieved by hypodermic injection of morphia.

*Strychnia in the advance stages of Typhoid Fever.*—Prof. N. S. Davis states (Chicago Medical Examiner,) that he knows of no remedy as direct and efficient for sustaining the nervous functions of animal and organic life as strychnia. In the advance stages of typhoid fever, when the muscular contractions of the heart become weak, and the sphincter muscles give indications of relaxation, he had for many years resorted to the use of strychnia with great advantage. He generally gives it in connection with nitric acid, and when the bowels are moving frequently, adds tr. opium:

| | | |
|---|---|---|
| ℞—Strychniæ, | . . . . . | gr i. |
| Nitric acid, | . . . . . | ʒ i. |
| Tinct. opii, | . . . . . | ʒ iij. |
| Aquæ, | . . . . . | ℥ iij. M. |

S. One teaspoonful every four hours. He also orders turpentine and laudanum emulsion to be given between.

*Carbolic Acid in Flavus and Crusta Lactea.*—Dr. F. K. Bailey recommends (Nashville Journal of Medicine and Surgery,) the following formula for these diseases:

| | | |
|---|---|---|
| ℞—Hyposulphite Sodæ, | . . | ℈j. |
| Carbolic acid, | . . | grs v. |
| Aquæ, | . . | ℥ i. M. |

S. Use to eruptions three times a day. Should the stools be green and slimy, and bowels disordered, he gives the one-twentieth of a grain of proto-iod. mercury, morning and night.

*Chronic Rheumatism.*—Prof. N. S. Davis (Chicago Medical Examiner,) employs the following formula in chronic rheumatism:

| | | |
|---|---|---|
| ℞—Vin. colch. sem. | . . | ℥ j. |
| Tinct. aconite rad. | . . | ʒ j. |
| Tinct. stramonii, | . . | ℥ ss. |
| Syrup and water, | . . | ℥ iss. M. |

S. Teaspoonful every four hours. In order to procure rest, if necessary, he gives fifteen grains of bromide of potassium at bed time.

*Staphisagria.*—Two drachms of the bruised seeds of the stavesacre digested in one ounce of melted lard, and strained while hot, furnishes an ointment which is superior to all other applications for destroying lice.—[Dr. Ringer's Therapeutics.—American Practitioner.

*Bromide of Potassium in Nettle Rash.*—Dr. McCall Anderson states that in genuine cases of urticaria perstans, recurring from day to day, and from week to week, when not dependant on disorder of the digestive organs, and independent of local causes are frequently cured by bromide of potassium.

*Camphor.*—When the mucous membrane of the nose, frontal sinuses, etc., is affected by catarrh, a strong solution of camphor frequently and for some hours snuffed up the nose, and five or six drops taken internally on a lump of sugar at first for every ten minutes, then every hour, will usually put a stop to the affection. Ordinary cold and even influenza, if treated in this manner at the *very beginning* of the attack, are generally controlled by the same treatment.

Attacks of incessant sneezing and profuse running of the eyes and nose will generally yield to a strong solution of camphor diligently snuffed up the nose. In summer diarrhœa no remedy is so efficacious as camphor, if employed at the very commencement of the disease; later it is without effect. Our author thinks its influence over cholera equally conspicuous, testifying to its almost magical effects in this disease. Dose: six drops of a strong alcoholic solution of camphor, given at first every ten minutes; afterward, as the symptoms abate, less frequently.—*Am. Prac.*

*Aconite.*—This is one of the most valuable drugs we possess. Its power over inflammation is little less than marvellous. In tonsilitis, catarrh of children, and acute sore-throat, especially if given the very moment the disease sets in, it generally relieves in from twenty-four to forty-eight hours. All the slighter forms of inflammation may be equally well treated by aconite. The urgent dyspœna and fever of catarrhal croup are conspicuously under its control. Pnenmonia, pleurisy, and the graver inflammations may be considerably curtailed and made milder by aconite. The remedy should, if possible, be given at the very beginning of the disease. Every hour is of importance. The dose is half a drop or a drop of the tincture, given in a tea-spoonful of water, every ten minutes for two hours, and afterward every hour. If the temperature, as indicated by the thermometer be natural, the case is not one for aconite. In the secondary fever which sometimes follows on the exanthems, aconite is particularly efficacious.—*Ib.*

*Digitalis.*—In dilated heart, accompanied by feeble, frequent fluttering and irregular pulse, digitalis may be given with the confident expectation of relief. The irregularity of the pulse constitutes the most reliable indication for the use of the drug. Our author uses the infusion. One or two drachms of this taken twice or thrice a day is often of signal service in spermatorrhœa, in the paroxysmal dyspnœa of bronchitis dependant upon palpitation of the heart, and in the palpitations and oppression of breathing witnessed in the subjects of dilatation, with hypertrophy of the left ventricle.—*Ibid.*

*Varicose Ulcers.*—Dr. Geo. D. Stanton highly extols (Med'ı and Surgical Reporter) a poultice made of the Dirca palustus—wicopy, leatherwood, rope-bark—for varicose ulcers. The inner bark is scraped off by a dull knife—a portion of this is made into a poultice, with boiling water, and applied to the ulcer and allowed to remain twenty four hours and replaced every day. He uses also cit. ferri et quin. as a tonic, and opium to allay pain.

*Chloral in Cerebro-Spinal Meningitis.*—Dr. H. B. Robinson, Forkland, Greene county, Alabama, in an article furnished the Medical Gazette, says meningitis prevailed in his vicinity during the winter of 1864 and 1870, of a very fatal character. He used every method of treatment recommended by writers—antiphlogistics, mustard-baths, fly-blisters to extremities, quinine, etc. In a few cases used opium with better success than the preceding remedies. He also gave mercury, bromide of potassium and morphine. He found at a later period better results from the use of chloral, keeping the patient continuously under its influence. He remarks that were he now called to treat meningitis, he would go forth with less forebodings than heretofore, of the result. He would first endeavor to free the patient from pain as speedily as possible, by giving chloral and morphia to be continued until relief was had, at the same time giving the richest nourishment, leaving the inflamation to nature and time. We have before noticed the value of chloral in this disease. Dr. D. W. Hammond, of Macon has used it successfully in one case reported in the May number of this Journal. Chloral was suggested by the writer (Jr. Ed.) two years ago as a remedy for the disease upon theoretical grounds.

*Bromide of Iron in Tertiary Syphilis.*—Dr. N. A. Norris, (Northwestern Medical and Surgical Journal,) says: "In this disease it is fully equal to the iod. potass., and in my hands it has cured some where the iod. failed. It has been my practice for the past two years to alternate the iod. pot. with the brom. ferri, in the treatment of spyhilis in its third stage."

*Nitro-Muriatic Acid in Urticaria.*--A. M. Lyles, M. D., of Early Grove, Mississippi, writes that he has found, for many years past, that ten drops of nitro-muriatic acid, in a wine-glassful of water, one hour before eating, is an almost unfailing remedy for urticaria. He has observed that the disease is attended by the generation in the alimentary tube of an excessive amount of acid, which the nitro-muriatic acid, given as above, corrects.

*Colocynth.*—Two or three drops of the Prussian tincture on a lump of sugar, given three or four times a day, will usually relieve constipation.—*American Practitioner.*

*Chloral in Obstetrics.*—Dr. S. F. Starley, of Fairfield, Texas, (Am. Practitioner,) has for nearly twenty years used chloroform in parturition, but has now substituted chloral for it, finding it a valuable anæsthetic, and in many respects greatly preferable to chloroform. He usually commences with 20 grains; in one or two hours, 15 to 20 grains again, and then enough to keep up the anæsthetic effect. He has found it hignly valuable in puerperal convulsions.

*Spongio-Piline.*—This is the name of an ingenious contrivance, recently introduced abroad, which may be used either as a poultice or as the means of fomentation. It consists of wool and small particles of sponge, apparently felted together and attached to a skin of india-rubber. It is about half an inch in thickness. It retains heat for a considerable time, and by means of vinegar, laudanum, camphor, hartshorn, etc., can be placed on the skin, readily, and with greater cleanliness than by the use of ordinary poultices.—*Boston Journal of Chemistry.*

*Cure of Fistula in Ano without the Knife.*—Dr. E. C. Huse, Rockford, Ill., gives (Medical Record) his method of treating fistula in ano. He uses the ordinary hypodermic syringe, with small silver canula, which readily takes the shape of the fistulous track, through which he injects a *saturated etherial tincture of iodine.* He first explores the fistula with a *very small* probe (the ordinary probe being too large,) in order to determine its course and extent. The patient being then placed in a good light, the glass rectal speculum is introduced, with its fenestrum opposite the internal orifice of the fistula. The canula is bent to the required curvature, and introduced, the syringe then screwed on and the surface thoroughly cleansed with tepid water. The fistula is then by pressure its entire length dried out. He then introduces in the speculum a quantity of carded cotton to absorb the excess of injection. The canula is re-inserted, and the injection *slowly* made, while the canula is being gradually withdrawn, thus carrying the iodine to every part of the fistulous track. The operation is not painful and the patient need not be confined to his bed or room for even an hour.

*Hypodermic injection of morphia in cholera morbus and dysentery.*—Dr. Thomas J. Gallaher, Pittsburg, Penn., has employed morphia hypodermically with marked success in the above diseases. He gives (N. Y. Medical Journal,) a number of cases. In one case he injected the morphia on account of the abdominal pain, and was astonished to find that it relieved the vomiting and purging as well. In subsequent cases he took advantage of the knowledge thus obtained, and injected the morphia for all the symptoms, omitting all other remedies, even the mustard plaster so generally employed. The success was beyond anything hitherto observed by him. The diarrhœa and vomiting ceased at once, and in four out of five the pains and cramps also. During last summer he treated three cases of dysentery with complete success, the pain and tenesmus being instantly relieved. The cure is more rapid than by any other treatment. He uses only one or two injections (mostly but one) daily. He has also resorted to this method in sêmi-chronic forms of dysentery and diarrhœa with entire success.

*Dysentery treated by Ipecac.*—Dr. MacCallum reports four cases of dysentery (Canada Med. Jour.) treated with large doses of ipecac. In every case the success of the treatment was most marked and flattering. Twenty grains was the maximum dose given, and it always followed a dose of tr. opii gtt 15 given one hour before, and with express instructions that no more than a teaspoonful of fluid was to be given at a time; by this means the tendency to vomit was lessened.

*Iodoform Ointment.*—In the Boston City Hospital iodoform ointment, in connection with iodide of potassium, is extensively and successfully used in the treatment of syphilitic ulcers and rupia. Dr. Wm. Ingalls, attending surgeon, advocates this formula:

℞—Iodoformi, . . . . ʒ ss.

Spts. vini rect. . . . q. s.

Adipis suillæ, . . . ʒ vijss. M.

*Ascites successfully treated by oil of copabia.*—Dr. Sieverking (London Lancet) in a case treated in St. Mary's Hospital, gives the following history: The patient's abdomen began to enlarge after an attack of diarrhœa, with pain over the region of the spleen. Had intense pain, the abdomen being greatly distended, shiney and dull on percussion; superficial vein congested, and skin hot and dry; tongue furred; pulse 94; urine scanty, high-colored but not albuminous. Mercurials, salines, with iodide potassium, iron and an occasional opiate, together with leeches, poultices and blisters, were tried, but the infusion increased, the abdoomen measuring 39 inches round the umbilicus. He then ordered ten minims each oil copabia and liquor potassæ, in an ounce of camphor mixture, three times a day. The patient improved, and under the iron and quinine mixture, as a tonic, was discharged quite well after some weeks.

*Nux Vomica.*—In chronic catarrh of the stomach, one or two drops of the tincture of nux vomica, taken every one or two hours, will usually, in the space of a day or two, clean off the tongue, and improve the appetite and digestion. In acute gastric catarrh, accompanied by sick headache, a drop of the tincture in a tea-spoonful of water, taken every five or ten minutes for an hour or two, and then at longer intervals, often gives quick relief. Weight at the pit of the stomach, acidity, heartburn, and flatulence after food, to which are added heat and weight at the top of the head, can generally be at once removed by five drops of the tincture, taken fifteen minutes before eating. The heat and feeling of weight on the top of the head, when independent of gastric trouble, are almost invariably relieved by the same treatment. One or two drops of the tincture twice or three times a day is amply sufficient with most persons suffering from constipation to insure one comfortable motion. Where the constipation is due to too small a supply of bile, as shown by the pale color of the stools, nux vomica is insufficient. The tincture, especially when combined with small quantities of laudanum, is particularly useful in the so-callod hysteria met with in middle-aged people.–*Am. Prac.*

*Lobelia.*–-This medicine is better borne by children than by adults. Our author gives to children, two years old, with pertussis, the tincture in doses of ten minims every hour, and an additional dose each time the cough comes on. The remedy has the most remarkable power over the cough, but it is not certain that it diminishes the duration of the disease.—*Ibid.*

*Cannabis Indica.*---Dull throbbing pain of the head, situated over one brow, and around the eye, most generally of the right side, lasting from a few minutes to several hours, sometimes attacking the muscles at the back of the neck, and leaving the skin very sore and tender, will speedily yield to the tincture of Indian hemp, taken in doses of three drops every four hours.---*Ibid.*

*Diagnosis of pain, whether rheumatic or neuralgic.*—Dr. J. M. Fothergill remarks that rheumatism is always accompanied by the presence of an acid both in the secretions and excretions. He prefers testing the saliva for acid rather than the urine—the former in health being always alkaline during digestion. If, therefore, litmus paper be reddened by it, decidedly, during digestion, we may say, almost surely, that it is caused by excess of acid, and therefore pain occurring under such circumstance, is due to rheumatism. On the contrary, when the reaction is not acid, the complaint is essentially neuralgic, and a clue is at once furnished as to the proper treatment. Where there is decided acidity, relief is always afforded by alkalies. Growing children often have an acid condition of the system, due to uric acid (as well as strumous adults,) and in such cases potash acts as a virtual hæmatic and blood restorative. Potash is the remedy for an excess of uric acid; soda is just as useful for an excess of lactic acid.

